# Factor B Inhibition with Iptacopan in Recurrent C3 Glomerulopathy Following Kidney Transplant: A Report of Two Cases

**DOI:** 10.1016/j.xkme.2024.100823

**Published:** 2024-04-12

**Authors:** Víctor J. Escudero-Saiz, Ángela Gonzalez, Adriana García-Herrera, Ana B. Larque, Andrew S. Bomback, Laura Morantes, Marta Martínez-Chillarón, Júlia Ollé, Elena Guillén, Marc Xipell, Alicia Molina-Andújar, Diana Rodríguez, Elena Cuadrado, Judit Cacho, Carolt Arana, Núria Esforzado, Carla Bastida, Esteban Poch, Fritz Diekman, David Cucchiari, Luis F. Quintana, Miquel Blasco

**Affiliations:** 1 Nephrology and Kidney Transplantation Department, National Reference Center on Complex Glomerular Disease (CSUR), IDIBAPS, Hospital Clínic, University of Barcelona, Barcelona, Spain; 2 Pathology Service, Hospital Clínic Barcelona, Barcelona, Spain; 3 Division of Nephrology, Department of Medicine, Columbia University Irving Medical Center, New York, NY; 4 Pharmacy Department, Division of Medicines, Hospital Clínic, University of Barcelona, Barcelona, Spain

**Keywords:** C3 glomerulopathy (C3G), transplant recurrence, kidney graft loss, complement system, alternative complement pathway, factor B inhibition, iptacopan

## Abstract

C3 glomerulopathy is a rare disease caused by fluid phase dysregulation of the alternative complement pathway. Currently, treatment depends on clinical and histological severity and includes nephroprotection, unspecific immunosuppression, and terminal complement blockers (C5), without having an etiological treatment approved. C3 glomerulopathy has high recurrence rates after kidney transplantation with a high risk of graft loss. Fortunately, new molecules are being developed that specifically target the proximal alternative complement pathway, such as iptacopan, a factor B inhibitor that showed promising results in native kidneys and cases of transplant recurrence in a phase 2 clinical trial. We present 2 “real-world” cases of C3 glomerulopathy recurrence in kidney allografts treated with iptacopan, with initial excellent clinical response and safety profile, especially with early introduction. We also present follow-up biopsies that showed no C3 deposition during factor B inhibition. Our cases suggest that proximal blockade of the alternative complement pathway can be effective and safe in the treatment of C3 glomerulopathy recurrence in kidney transplantation, bringing other questions such as dual blockade (eg, in C3 and C5), the optimal patient profile to benefit from factor B inhibition or treatment duration and its potential use in other forms of membranoproliferative glomerulonephritis (eg, immune complex-mediated).

## Introduction

C3 glomerulopathy (C3G) is a rare disease caused by fluid phase dysregulation of the alternative complement pathway (ACP). The hallmark histologic finding is intense C3 deposition, exclusively or greater than that of other immunoreactants, on immunofluorescence microscopy.^1^ Currently, an etiological treatment for C3G is unavailable. Standard of care involves supportive therapy (antihypertensive and antiproteinuric drugs) and nonspecific immunosuppression with corticosteroids and mycophenolate mofetil (MMF). Terminal complement inhibitors have been tested in refractory cases or crescentic forms with varying success.^2^

The recurrence risk of C3G after kidney transplant is at least 70%.^3^ Predisposing factors include disease severity and young age at onset, decreased serum C3 levels (sC3), and increased serum membrane attack complex levels.^3^ Despite some favorable results with eculizumab,^4^ no treatment has demonstrated efficacy after C3G recurrence in kidney transplantation.

New molecules targeting different ACP components are under development for C3G treatment both in native and transplanted kidneys. Iptacopan targets factor B (FB) in the fluid phase of the ACP.^5^ FB interacts with the serine protease domain (Bb), which is the active component of the C3 and C5 convertases. Therefore, iptacopan suppresses C3 convertase activity and holds forth promise as an effective therapy for C3G in both scenarios; early results from a phase 2 trial appear to support this hypothesis.^6^ We present 2 “real-world” cases of early C3G recurrence after kidney transplantation successfully treated with iptacopan.

## Case Report

### Case 1

A man in his early 30s presented with nephrotic syndrome and hematuria. Kidney biopsy at that time revealed C3G. Laboratory tests showed low sC3 and the presence of C3 nephritic factor. Treatment with angiotensin II receptor antagonist and oral corticosteroids (0.5 mg/kg/day), along with MMF (500 mg/8 hours), resulted in a partial response. Genetic testing revealed a variant of uncertain significance in *C3* (heterozygosity in exon 24 c.3023C>T) and deletion of *CFHR3* and *CFHR1* in heterozygosity. Later, nephrotic-range proteinuria and hematuria relapse prompted addition of rituximab (375 mg/m^2^ × 4 doses), along with increasing MMF (3 g/day) and oral cyclophosphamide (150 mg/day). The patient did not respond and required hemodialysis (HD) 19 months after disease onset.

**Figure 1:**
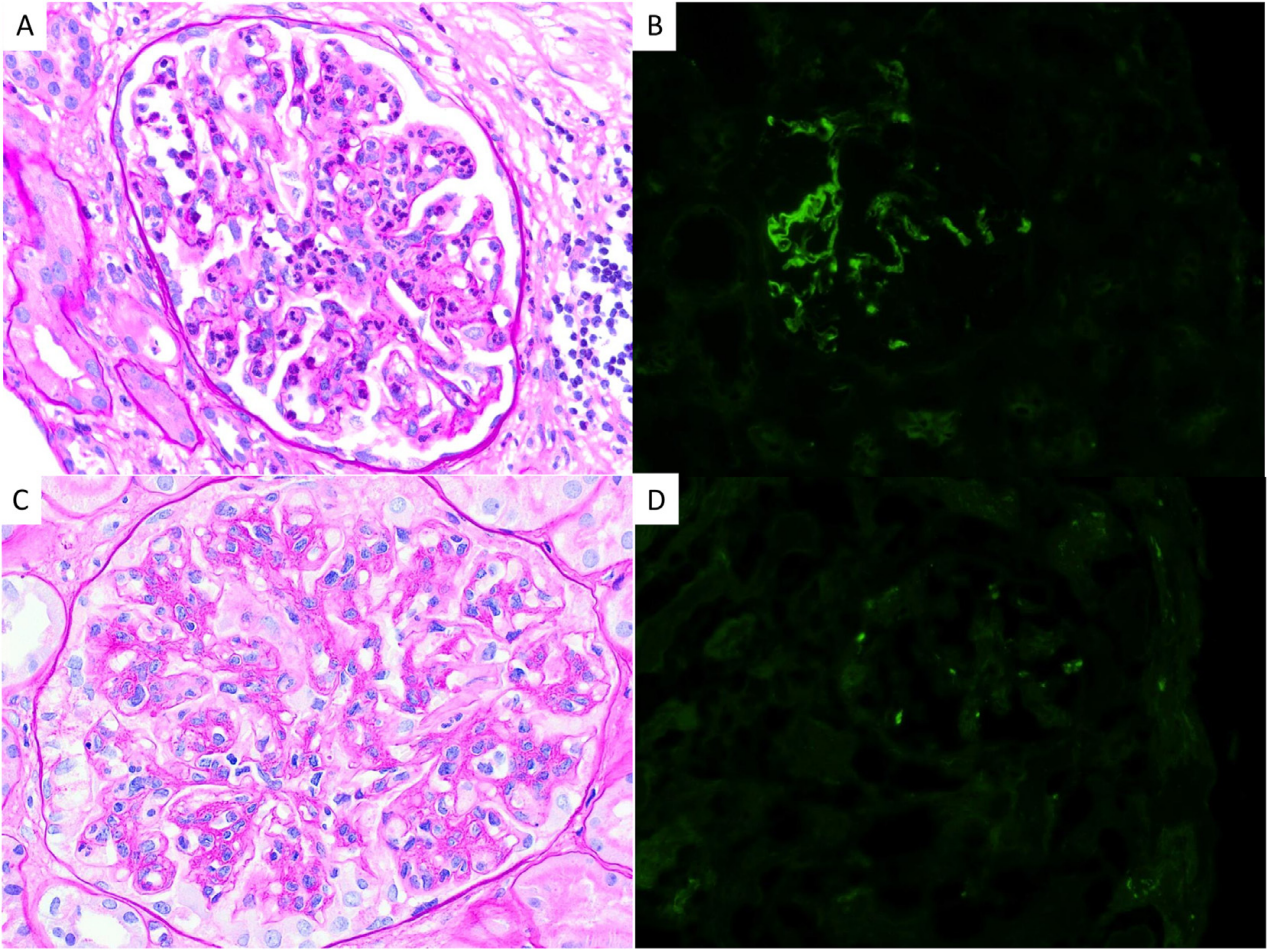
Case 1: kidney transplant biopsies. Superior: first biopsy with C3 glomerulopathy recurrence diagnosis. (A-B) Light microscopic image showing endocapillary proliferation (A), periodic acid–Schiff (PAS). (B) Immunofluorescence staining with strong C3 deposition. Inferior: biopsy control after 5 months of initiation of iptacopan. (C-D) Light microscopic image with persistent endocapillary proliferation with mesangial proliferation and presence of double contours pattern (E), PAS. (D) Immunofluorescence with C3 impregnation but no significant deposits. Original magnification, 100× in A, B, C, and D.

The patient was admitted 5 years later as a kidney transplant recipient from deceased donor (Maastricht III). Induction was based on thymoglobulin and maintenance on tacrolimus, MMF, and corticosteroids. Kidney allograft biopsy 1 week after transplantation because of delayed graft function revealed acute tubular necrosis, endocapillary proliferation, and dominant C3 deposition (+++), without C4d and with unspecific C1q and IgM deposits (+/− for both) (Fig 1A and B). The patient was treated with corticosteroid pulses, a single dose of rituximab, and 5 plasma exchanges, with no response. Subsequent biopsy, while still HD dependent, revealed persistent C3G and improved acute tubular necrosis, with intense mesangial C3 deposits (+++/+++) and C1q (+/+++) but no other immunoreactants.  Weekly renal scintigraphy suggested improvement of ATN, with counts per second increasing from 267 to 630 and appearance of an excretory phase. Given the apparent improvement in ATN despite ongoing hemodialysis dependence, we hypothesized that C3G recurrence was the primary cause of DGF; accordingly, we initiated iptacopan (200 mg/12 h oral) through Novartis’ Managed Access Program (MAP) after the patient signed a consent form.

After 3 weeks, kidney function improved enough to discontinue HD (Fig S1A). There was also an increase in sC3 to 0.3 g/L and a reduction in proteinuria to 1.6 g/g (Fig S2A). Histological examination at 5 months showed no deposition of C3 or other immunoreactants and a shift from acute proliferative to membranoproliferative pattern of the lesion (Fig 1C and D). However, after an unintentional 5-day treatment discontinuation, he experienced clinical recurrence with hematuria and acute kidney injury requiring HD, which resolved on iptacopan restart. After 12 months of anti-FB therapy, kidney function stabilized with serum creatinine level of 2.5-2.6 mg/dL and estimated glomerular filtration rate of 28-30 mL/min/1.73 m^2^.

### Case 2

A woman in her early 40s was diagnosed with C3G by kidney biopsy at the beginning of adulthood (nephrotic syndrome and hematuria). She had a partial response to angiotensin II receptor antagonist, corticosteroids, MMF, and tacrolimus. After 20 years, a relapse occurred, and a second biopsy showed C3G associated with thrombotic microangiopathy. Blood analysis revealed low sC3 and anti-factor H antibodies. Genetic testing showed 2 *diacylglyceron kinase-epsilon* (*DGKe*) variants (heterozygosity in exon 2 c.117G>C and exon 10 c.1359C>T). Despite eculizumab (900 mg × 4 doses, later 1,200 mg/15 days) and rituximab (375 mg/m^2^ × 4 doses) administration, HD was initiated 4 months after relapse.

**Figure 2:**
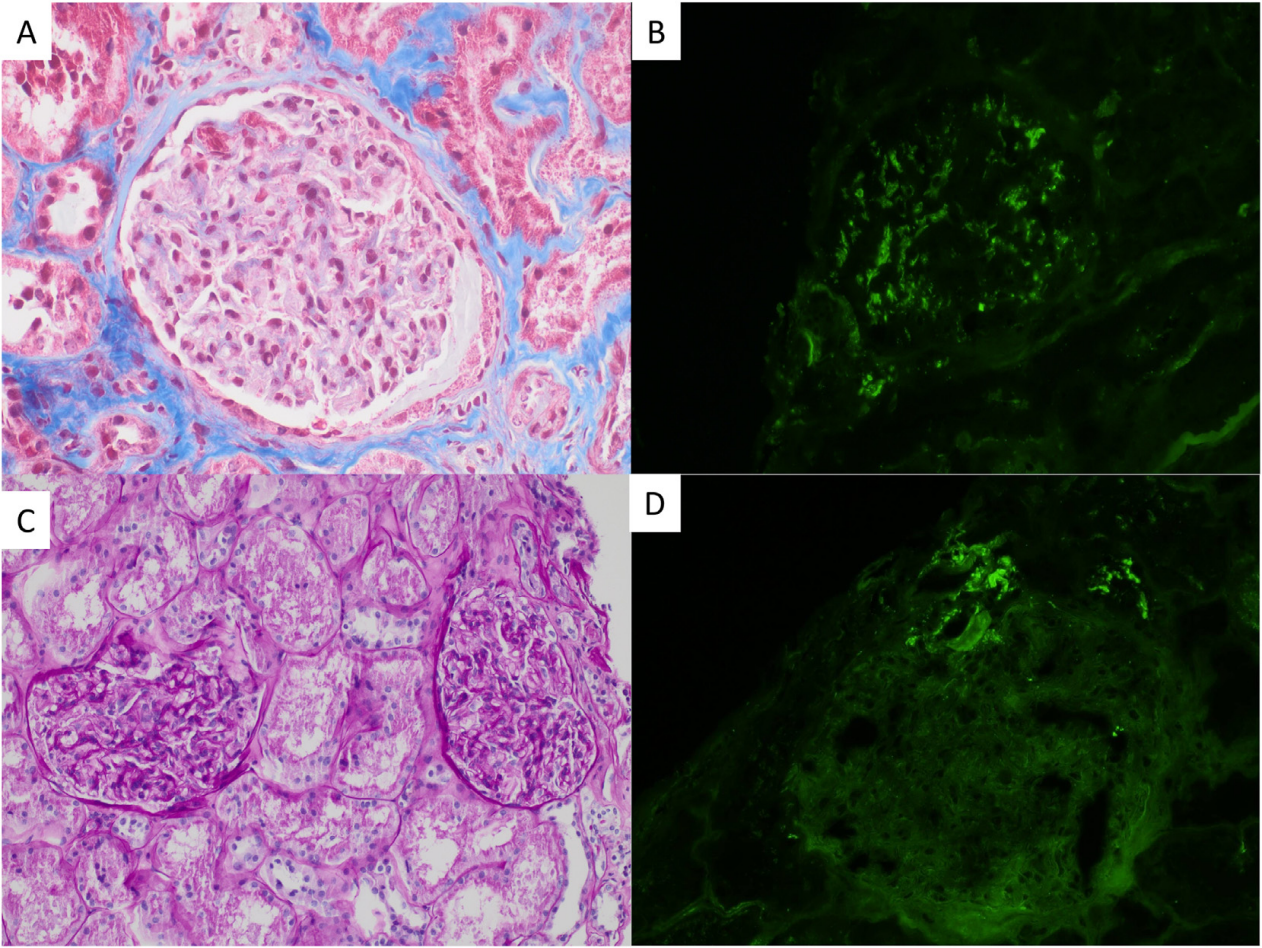
Case 2: kidney transplant biopsies. Superior: first biopsy with compatible initial signs of C3 glomerulopathy recurrence. (A) Light microscopic image showing mild expansion of mesangium with slight hypercellularity, Masson’s trichrome. (B) Immunofluorescence with C3 deposition in the mesangium (++). Inferior: biopsy control after 8 months of the initiation of iptacopan. (C) Light microscopic image with normal glomeruli with no hypercellularity in any compartment, periodic acid–Schiff. (D) Immunofluorescence staining with no C3 deposition in the glomeruli (vascular pole deposit as internal control). Original amplification, 100× in A, B, C, and D.

The patient was admitted 8 months later for a living-donor kidney transplant. Kidney graft evolution was satisfactory with induction based on basiliximab, tacrolimus, MMF, and corticosteroids. An early posttransplant biopsy specimen showed no significant mesangial C3 deposits (++/+++) without cellular proliferation or positivity for other immunoreactants. A mild increase in proteinuria prompted a second allograft biopsy 4 months after transplantation, which revealed an incipient C3G recurrence by the presence of mesangial C3 deposits (++/+++) and unspecific C1q deposits (+/−) with slight mesangial proliferation (Fig 2A and B). Consequently, the patient began iptacopan (200 mg/12 hours oral) through Novartis’ Managed Access Program after signing a consent form.

After 8 months, a control biopsy showed disappearance of C3 deposits and other immunoreactants and no hypercellularity in any glomerular compartment (Fig 2C and D). Serum creatinine level remained stable at 1.3-1.4 mg/dL with an estimated glomerular filtration rate of 45-50 mL/min/1.73 m^2^ (Fig S1B). This was accompanied by proteinuria decrease and increase in sC3 reaching normality (Fig S2B), despite persistent positivity for anti-factor H antibodies. No treatment discontinuation or clinical recurrence was observed.

In both cases, proper vaccination was administered for *Neisseria meningitidis, Streptococcus pneumoniae*, and *Haemophilus influenzae,* as in the phase 2^6^ and phase 3^7^ studies with iptacopan in C3G. There were no infections reported.

## Discussion

We present 2 “real-world” cases of C3G recurrence after kidney transplantation treated successfully by compassionate use of iptacopan, an FB inhibitor. These cases represent a clinical spectrum of disease ranging from a living-donor kidney transplant recipient with incipient histological damage to a deceased-donor transplant with extensive histological damage associated with C3 deposits. In both cases, a good clinical response was obtained with a common histological finding of resolution of the C3 deposits, after specific blockade of the ACP in the fluid phase by FB inhibition.

C3G recurrence after kidney transplantation is frequent, reported in 60%-80% of cases,^8^^,^^9^ with attributed graft loss rates from 50%-80%.^8^^,^^10^ Gonzalez Suarez et al^11^ compiled 122 C3G recurrence cases reported in the literature, showing lower graft loss rates in those treated with eculizumab (33%) than plasma exchange (42%) or rituximab (81%). Serum membrane attack complex level was elevated in 80% of eculizumab responders, consistent with the first reports of successful eculizumab therapy in C3G from Bomback et al.^12^ Remarkably, 66 cases with histological recurrence were untreated because they had no clinical manifestations, with 40% graft loss rate, higher than the eculizumab group (33%). Le Quintrec et al^2^ presented their experience with eculizumab in 26 C3G cases in native kidneys with remission rates of about 46% (23% complete and 23% partial), concluding it was effective in rapidly progressive forms and/or those with extracapillary proliferations in the biopsy specimens. However, C3G recurrence treatment is still controversial because of unavailable substantial evidence supporting the benefit of eculizumab (absence of randomized trials including control arms). None of our cases had crescents in their first biopsy specimen following C3G recurrence, and in Case 1, there were 2 possible causes of delayed graft function evolution (in the absence of extracapillary proliferation), so we decided to avoid eculizumab. Currently, there are several ongoing clinical trials investigating the use of new complement inhibitors targeting C3 level in various glomerulopathies, including C3G^13^ (Table S1), as a dysregulated ACP is the underlying pathogenic driver of C3G.

Based on the disease severity in the native kidney and absence of response to conventional therapies, we decided to initiate compassionate use of iptacopan in our patients. We observed a partial response in Case 1, allowing HD discontinuation, and complete response in Case 2 with proteinuria reduction. Both patients could reach estimated glomerular filtration rate stabilization, in accordance with the results of the phase 2 study of iptacopan in C3G in native kidneys.^6^ Importantly, we found no C3 deposition in the follow-up biopsy specimens, similar to that reported in the transplanted arm of the abovementioned study, with a reduction in C3 deposition after almost 3 months of treatment, despite the persistence of a glomerular inflammation pattern in Case 1.

Furthermore, the clinical response after iptacopan discontinuation in Case 1 was significant, with acute kidney injury requiring HD and hematuria. Resuming iptacopan restored kidney function to previous values. Throughout the follow-up, the patients did not experience any adverse drug reactions or encapsulated bacterial infections. Both patients received proper vaccinations before drug initiation.

These 2 cases highlight several fundamental aspects when currently addressing patients with C3G who require a kidney transplant. First, the potential role of therapy directed against the ACP with new molecules that target higher up in the complement pathway at the C3 level is an important step in the evolution of C3G treatment. Iptacopan has shown favorable safety and efficacy profiles in a phase 2 study^6^ with a large phase 3 study now underway.^7^ Second, effective therapies against C3G in the native and allograft kidney will likely have the greatest impact if used at the earliest signs of disease onset and for allograft kidneys; this will be best detected by protocol biopsies as in our second case. Finally, we highlight that living-donor kidney transplant may provide remarkable advantages for recipients with C3G, such as less risk of rejection and immediate allograft function, which can minimize the effect of an early C3G recurrence, like in Case 2. However, there are still unanswered questions, such as whether complement blockade could be included in induction therapy to prevent recurrence or saving it until this is detected and to determine which patient profile could benefit most, as well as determining treatment duration. Additionally, it remains unclear whether dual blockade at both the C5 and C3 levels could be superior to blocking just one complement step. Finally, if FB inhibition is effective for native and recurrent C3G, its potential use in other forms of membranoproliferative glomerulonephritis should be explored, particularly those associated with immunoglobulins.
