# Supplementary material for: Factor B Inhibition with Iptacopan in Recurrent C3 Glomerulopathy Following Kidney Transplant: A Report of Two Cases
Source: Kidney Med. 2024 Apr 12;6(6):100823. doi: 10.1016/j.xkme.2024.100823 (PMC11089394; doi:10.1016/j.xkme.2024.100823)

Table S1: Clinical trials investigating the use of new complement inhibitors in C3 Glomerulopathy

Clinical trial	Drug	Target	Results	Transplant population
NCT03301467 (phase 2)	Avacopan	C5a receptor	Improvement in:<br>- eGFR<br>- Proteinuria<br>- Histological chronicity index	Yes (2 patients)
NCT0383211 (phase 2)	Iptacopan	Factor B	1) Native kidneys:<br>- Drop 45% proteinuria.<br>- eGFR stabilization<br><br>2) Allograft: reduction of C3 deposition (even disappearance)	Yes (11 patients)
NCT04817618 (phase 3)			NA	Non-included
NCT04572854 (phase 2)	Pegcetacoplan	C3	NA	Yes (ongoing)
NCT03124368 (phase 2)	Danicopan	Factor D	NA	Non-included
NCT02682407 (phase 2)	Narsoplimab	MASP-2	NA	Non-included

Abbreviations: NA: non-available; MASP-2: mannan-binding lectin serine protease 2.

Figure S1. Kidney graft evolution in case 1 (A) and case 2 (B). Blue arrows: start of Iptacopan. Orange arrows: protocol biopsies. Red arrow: clinical relapse after iptacopan discontinuation in Case 1. Right blue arrow (star): restart of iptacopan in Case 1. Dark blue line: serum Creatinine (sCr). Green line: estimated glomerular filtration rate (eGFR).

A

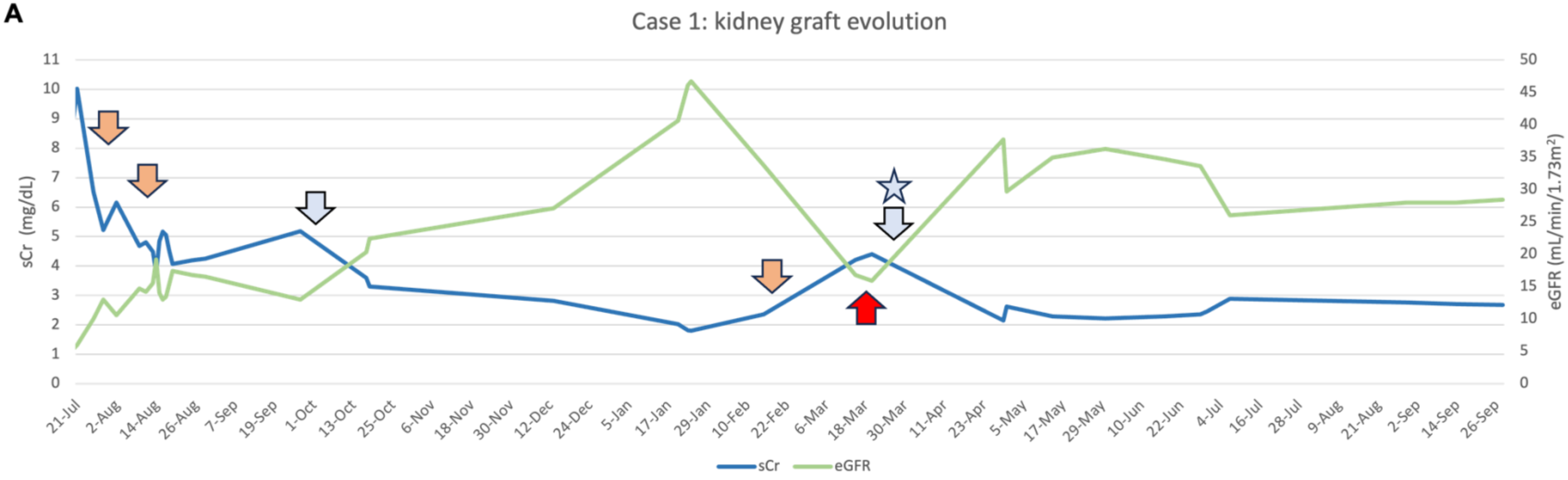

B

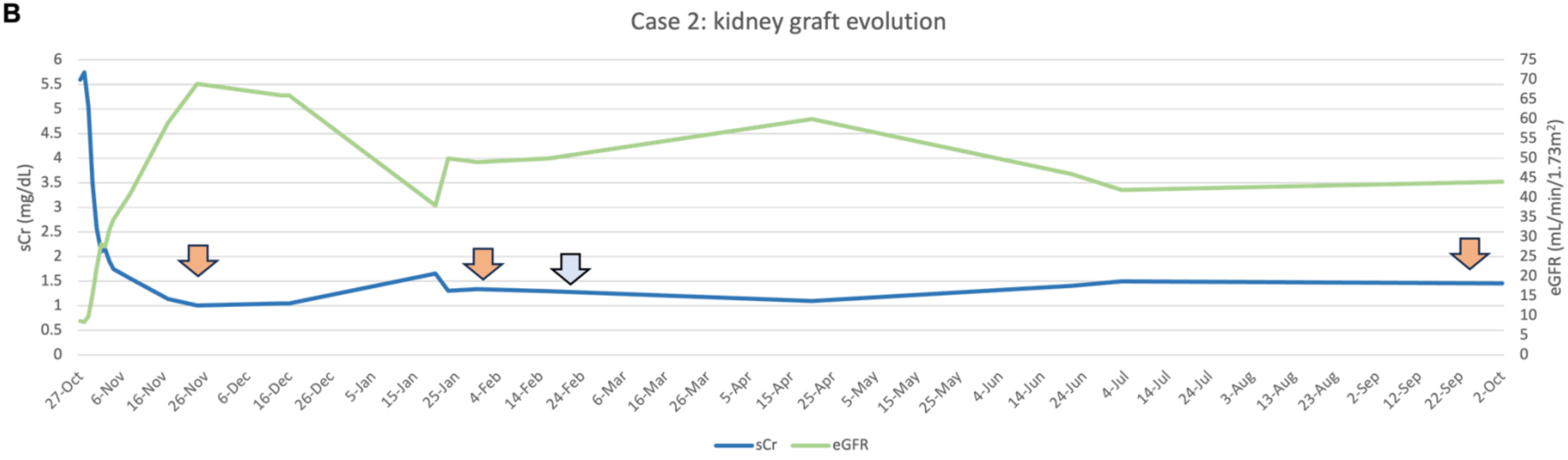

Figure S2. Proteinuria and serum C3 levels evolution in case 1 (A) and case 2 (B). Blue arrows: start of Iptacopan. Orange arrows: protocol biopsies. Red arrow: clinical relapse after iptacopan discontinuation in Case 1. Right blue arrow (star): restart of iptacopan in Case 1. Green line: serum C3 levels (sC3). Grey line: proteinuria in mg/g of creatinine.

A

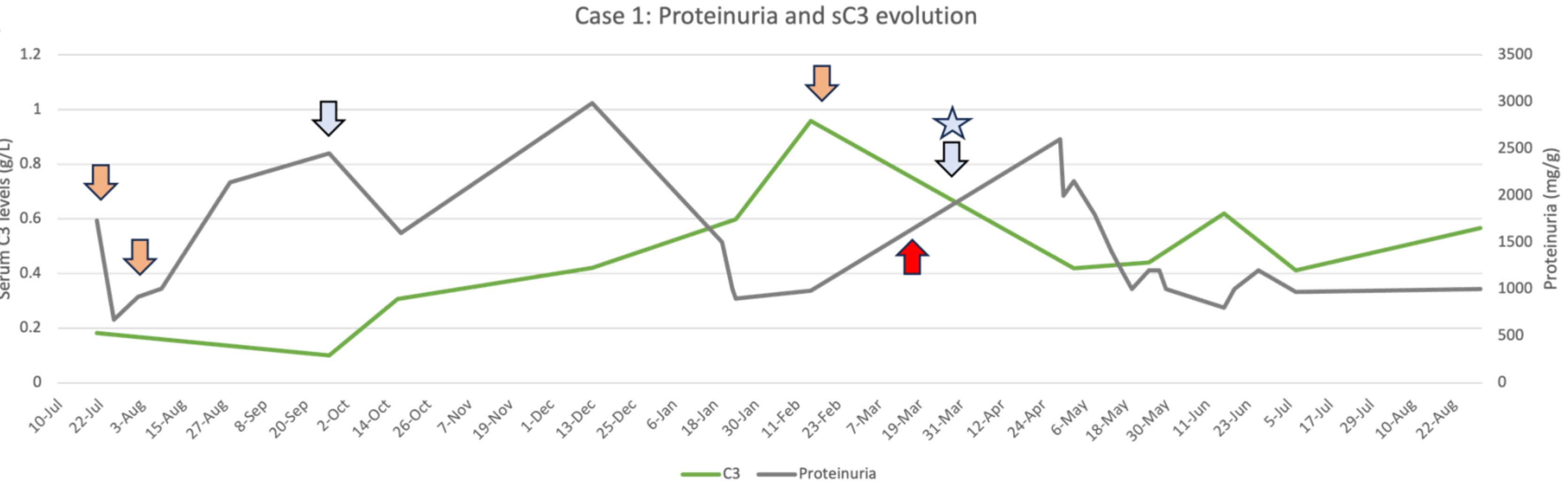

B

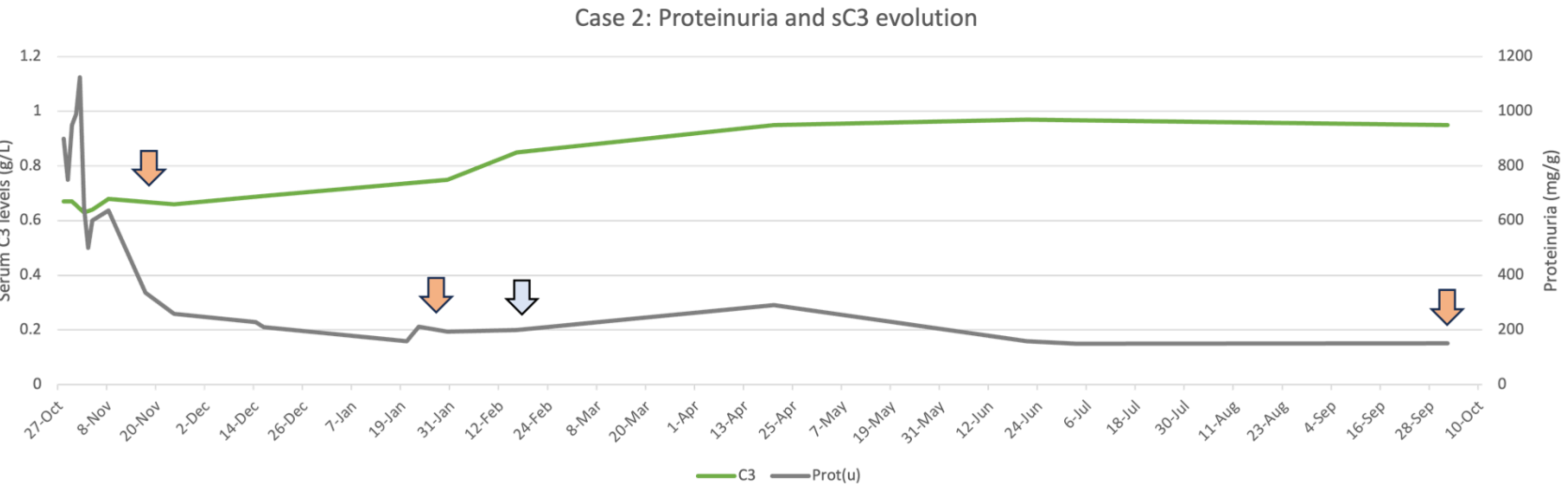

Supplement: Supplementary File (PDF) — Figure S1, Figure S2, Table S1. [file mmc1.pdf]
